# Commentary: Large Acid-Evoked Currents, Mediated by ASIC1a, Accompany Differentiation in Human Dopaminergic Neurons

**DOI:** 10.3389/fncel.2021.789354

**Published:** 2021-11-22

**Authors:** Matthew William, Som Singh, Xiang-Ping Chu

**Affiliations:** Department of Biomedical Sciences, School of Medicine, University of Missouri-Kansas City, Kansas City, MO, United States

**Keywords:** acid-sensing ion channel, dopamine cell, differentiation, patch-clamp recording, human cell

## Introduction

Acid-sensing ion channels (ASICs) are proton-gated Na^+^ channels widely located in the peripheral and central nervous system (Waldmann et al., 1997; Krishtal, 2015). They are often associated with neurological disorders involving a decrease in extracellular pH, such as cerebral ischemia and epilepsy (Xiong et al., 2008; Kweon and Suh, 2013; Li et al., 2016). Additionally, these channels also contribute to cognitive functions such as learning, memory and synaptic plasticity (Chu and Xiong, 2012; Huang et al., 2015; Gobetto et al., 2021). While the functions of ASICs are often explored in murine models, few studies have investigated these channels in human cells and human cell lines (Sun et al., 2020; Xu et al., 2021). Recently, a study conducted by the Grunder laboratory has examined the function of ASICs in human dopaminergic cell lines (Neuhof et al., 2021). They have proposed that ASICs are responsible for the differentiation of LUHMES (Lund Human Mesencephalic) cells, a line of human cells capable of differentiating into dopaminergic-like neurons (Zuberek et al., 2018; Harischandra et al., 2019). LUHMES cells are often used as a model to investigate cellular mechanisms of dopaminergic neurons in the substantia nigra (SN) and are used to further understand the pathology of disorders involving these neurons, such as Parkinson's disease (PD) (Zhang et al., 2014; Smirnova et al., 2016; Tüshaus et al., 2021). Their results ultimately reveal LUHMES cells to be a promising model in studying the role of ASICs in neuronal differentiation.

## Functional ASICs Modulate the Differentiation of LUHMES Cells

A recent study published in *Frontiers in Cellular Neuroscience* from the Grunder laboratory has explored the role of ASICs in the differentiation of LUHMES cells (Neuhof et al., 2021). They first converted LUHMES cells into post-mitotic dopaminergic neurons and subsequently isolated ASIC mRNA. They found that ASIC2 mRNA expression was profoundly lower than expression of ASIC1a mRNA, suggesting that ASIC1a is the dominant variant in LUHMES cells. Next, they measured the ASIC1a protein quantity and found that it was twice as high after 3 days of differentiation, however, any further increase in ASIC1a protein was halted after that. Whole-cell patch-clamp recording was used to record the ASIC currents. Peak ASIC current density started to increase significantly from day 0 to day 5, and then began to decrease over the next 5 days. These results demonstrate that a strong surge in ASIC current density exists within the first 5 days of LUHMES cell differentiation, confirming the presence of functional ASICs. Furthermore, the presence of homomeric ASIC1a in this cell line was largely examined by measuring ASIC current amplitude. Half maximal activation of ASICs was detected at pH 6.6 on day 4 and day 7 and pH 6.5 on day 5 and day 6, respectively, with a saturating amplitude of pH 6.0. These results strongly suggest that homomeric ASIC1a is likely responsible for the surge in ASIC current. Application of PcTx1, a selective ASIC1a inhibitor, resulted in complete inhibition of ASIC currents, further confirming the presence of homomeric ASIC1a. The presence of heteromeric ASIC1a/2b, however, cannot be excluded due to its similarities with homomeric ASIC1a, such as PcTx1 sensitivity. The presence of heteromeric ASIC1a/2a was not confirmed. Whether activation of ASICs can elicit action potentials (APs) in differentiating LUHMES cells was further investigated. At day 0, no APs were elicited upon depolarization, however, APs were elicited on day 5 and later after pH drop. The number of cells able to elicit APs nearly doubled from day 5 to day 10, indicating an increase in the excitability of LUHMES cells during the differentiation process. The role of ASICs in the physiological growth of LUHMES cells was further examined. After the ASIC pore blocker diminazene was applied, the length of neurites doubled from day 1 to day 5 of differentiation in a control population, while the diminazene-treated population contained shorter neurites on all days investigated. These results suggest that ASICs play a critical role in the neuronal differentiation process. Finally, the permeability of Ca^2+^ through ASICs was detected by using Fura-2 dye. Activation of ASICs by pH drop results in strong intracellular Ca^2+^ signaling. Upon application of the ASIC blocker amiloride, the Ca^2+^ response was nearly completely inhibited. While inhibition of voltage-gated Ca^2+^ channels by nimodipine was found to reduce Ca^2+^ signaling as well, it was not as strong as the reduction seen with the application of amiloride. Thus, activation of ASICs in LUHMES cells plays a dominant role in the intracellular Ca^2+^ signaling response.

## Discussion

The study conducted by the Grunder laboratory provides evidence supporting the role of ASIC1a in the Ca^2+^ homeostasis of LUHMES cells undergoing differentiation. As alluded to in the study, dysregulation in Ca^2+^ homeostasis is a contributing factor to the development of neurodegenerative disorders of dopaminergic neurons, such as PD. Oxidative stress is another risk factor involved in PD (Tabata et al., 2018; Trist et al., 2019). A recent study reported that ASIC1a was upregulated in NS20Y cells with exposure to H_2_O_2_ for at least 6 h (Wu et al., 2020). Thus, it is important to see whether the H_2_O_2_ treatment may upregulate ASIC1a expression in differentiated LUHMES cells. While H_2_O_2_ can induce oxidative damage to tissue which may also contribute to PD (Dehhaghi et al., 2018; Nandi et al., 2019), its role in potentially upregulating ASIC1a in dopaminergic neurons of the SN may contribute to overexpression of ASIC1a. The potential effects of this upregulation on calcium homeostasis in dopaminergic neurons may contribute to the calcium dysregulation hypothesized to cause PD (Mattson and Arumugam, 2018; Schrank et al., 2020). Therefore, investigation of the influence of H_2_O_2_ exposure on ASIC upregulation should be conducted to examine changes in intracellular calcium in LUHMES cells and cerebral dopaminergic neurons. Another avenue to explore is the limitations created by diminazene-treated LUHMES cells. While these cells have been shown to display physiological limitations, such as decreased neurite length by inhibition of ASICs, the ability to elicit APs or Ca^2+^ signals should also be investigated under ASIC blockers. If diminazene-treated cells with shorter neurites possess diminished or decreased ability to elicit APs or Ca^2+^ signals, this could provide further support for the importance of ASICs in LUHMES cell differentiation. A third avenue to investigate is the role of ASICs in disorders involving an increase in dopamine release, such as schizophrenia. The paper published by the Grunder laboratory suggests that ASIC1a may modulate dopamine secretion from human dopaminergic neurons (Neuhof et al., 2021). Schizophrenia is hypothesized to occur due to an over secretion of dopamine in the mesolimbic pathway (Birtwistle and Baldwin, 1998; Jones et al., 2011; Howes et al., 2017). Therefore, investigating ASIC1a in dopaminergic neurons of the mesolimbic pathway as a potential therapeutic target in limiting positive schizophrenic symptoms resulting from the over secretion of dopamine may prove clinically useful.

## Author Contributions

All authors listed have made a substantial, direct and intellectual contribution to the work, and approved it for publication.

## Funding

The work was supported by grant from American Heart Association (19AIREA34470007) to X-PC.

## Conflict of Interest

The authors declare that the research was conducted in the absence of any commercial or financial relationships that could be construed as a potential conflict of interest.

## Publisher's Note

All claims expressed in this article are solely those of the authors and do not necessarily represent those of their affiliated organizations, or those of the publisher, the editors and the reviewers. Any product that may be evaluated in this article, or claim that may be made by its manufacturer, is not guaranteed or endorsed by the publisher.
